# RETRACTION: Effect of Suture Cosure and Staple Closure on Postoperative Wound Complications in Patients Undergoing Knee Replacement Surgery: A Meta‐Analysis

**DOI:** 10.1111/iwj.70345

**Published:** 2025-03-18

## Abstract

**RETRACTION:** TangX. , ShiW. , QianY. , and GeZ. , “Effect of Suture Cosure and Staple Closure on Postoperative Wound Complications in Patients Undergoing Knee Replacement Surgery: A Meta‐Analysis,” International Wound Journal 21, no. 1 (2024): e14372, 10.1111/iwj.14372.37679956 PMC10782053

The above article, published online on 07 September 2023, in Wiley Online Library (http://onlinelibrary.wiley.com/), has been retracted by agreement between the journal Editor in Chief, Professor Keith Harding; and John Wiley & Sons Ltd. Following an investigation by the publisher, all parties have concluded that this article was accepted solely on the basis of a compromised peer review process. The editors have therefore decided to retract the article. The authors did not respond to our notice regarding the retraction.

**RETRACTION:** X. Tang , W. Shi , Y. Qian , and Z. Ge , “Effect of Suture Cosure and Staple Closure on Postoperative Wound Complications in Patients Undergoing Knee Replacement Surgery: A Meta‐Analysis,” International Wound Journal 21, no. 1 (2024): e14372, 10.1111/iwj.14372.37679956 PMC10782053

The above article, published online on 07 September 2023, in Wiley Online Library (http://onlinelibrary.wiley.com/), has been retracted by agreement between the journal Editor in Chief, Professor Keith Harding; and John Wiley & Sons Ltd. Following an investigation by the publisher, all parties have concluded that this article was accepted solely on the basis of a compromised peer review process. The editors have therefore decided to retract the article. The authors did not respond to our notice regarding the retraction.

